# The Book World of Medicine and Science

**Published:** 1902-05-31

**Authors:** 


### The Book World of Medicine and Science.

Recent Object Lessons in Penal Science: With a Bibliographical Introduction. By A. R. Whiteway, M.A., Barrister-at-Law. (London: Swan, Sonnenschein and Co., Limited. 1902 Pps. 212. Price 3s. 6d. net.)

Philosophers, from time immemorial, have laboured to devise means for the prevention of crime and all their suggestions may be considered to lie either at or between the extremes of an "eye for an eye" and the theory that no such thing as free will exists, so that each and every criminal must be regarded and treated as psychologically irresponsible. Mr. Whiteway believes in punishment in some few cases, but appears to be strongly inclined towards the latter school. Heredity as a predisposing cause is rather at a discount just now in all diseases due to bacteria, but is still rigorously insisted upon as an important factor in mental conditions. It is not, however, suggested in this volume that criminals should be prevented from being fathers and mothers, but rather that prisons should take more the form of hospitals, Mr. Whiteway having but little faith in the present system as a preventative of crime, especially in the case of *recidivists*. He asserts, and we think correctly, that more care should be taken to inquire into the mental condition of the prisoner before sentence, and that even greater care should be taken to provide suitable surroundings, diet, and occupation after conviction. The evils attending long incarceration before trial, and many practical suggestions to remedy this are ably stated, and attention is called to the prison officials, who are "too often inferior persons unsuccessful in other callings, who have taken to the prison service as a *pis aller*." But Mr. Whiteway not only quarrels with the subordinates, for he boldly states that "the judge is seldom what he should be—that is, a student of criminology"—and thinks that a mental expert, who "should be a man of science of the highest type and altogether different from the present prison doctor" should see the criminal every day to discover "his true characteristics and their most likely method of treatment." Many of us think that a professional psychologist who would no doubt label and classify each criminal as an interesting example of some form of mental aberration would neither make a good judge or a good prison doctor, for, in his enthusiasm as a specialist, he might be as liable to err on the side of leniency towards responsible persons as the present system possibly does in an opposite direction towards the irresponsible. The suggestion that prisons might be used "as cliniques for medical students desirous to enter the prison medical service" is a practical one, but such a special course of training would be more convenient for post-graduates. Mr. Whiteway is evidently unaware of the great difficulty of recognising early cases of general paralysis and of dementia or he would not choose instances of such diseases not being at once diagnosed to illustrate "the wretchedness of the state of affairs." The book as a whole is very interesting reading and is evidently the result of much careful study of the leading authorities on criminology.

The War against Consumption. By Dennis Vinrace, M.R.C.S., L.S.A.Eng. (The Century Printing Company, Limited. 1901. Pp. 178. 2s. 6d. net.)

This is a very useful summary of the speeches and papers delivered and read before the Congress on Tuberculosis. It is written in popular language, and all technicalities are carefully avoided, so that it should prove a valuable guide and help to all who are willing to co-operate actively in the campaign against consumption. Such practical measures as the disposal of sputum, the disinfection of houses, the provision of plenty of light and air are all insisted upon, and many useful hints are given to sanitary authorities who are willing to follow the praiseworthy example of the Council of the City of Manchester, whose methods are detailed at length. Mr. Dennis Vinrace shows both good taste and good sense in giving no opinion on Professor Koch's bolt from the blue, and recognising that the question is still *sub judice*, states the evidence for and against the theory that bovine and human tuberculosis are different diseases with absolute impartiality. Attention is drawn to the fact that tuberculin is a most valuable agent for diagnostic purposes in cattle and that it can also be safely used for the same purpose in human beings. Much is also claimed for the Rœntgen rays, which we hope will not lead to disappointment, for it must be remembered that the actual movements of the diaphragm in inspiration are not very easy to detect owing to the fact that the arched upper surface of the liver maintains its form, and that both the heart above and the spleen below on the left side also throw shadows. Every means which help to an early diagnosis and consequently lead to early treatment, must, however, be cordially welcomed and practised wherever possible.

Elementary Bandaging and Surgical Dressing. By the late Walter Pye, F.R.C.S. Ninth edition, revised by Thomas Carwardine, M.S. (Bristol: John Wright and Co. Pp. 214, 12mo. Price 2s.)

It is gratifying to note that, although new books are poured unceasingly from the press some old favourites still hold the field, and that amongst these latter we find Pye's "Bandaging," itself consisting mainly of excerpts from the older "Surgical Handicraft," and now revised by Mr. Carwardine, of Bristol. Many alterations and additions have been made, we notice, but there are still a few which Mr. Carwardine will, we doubt not, make in future editions. The account of the methods of using the triangular bandage leaves something to be desired; the "broad" and "narrow" fold bandages are not mentioned, nor the lesser arm sling. There is no description of Lister's famous T-turn chest bandage, and none of the well-known St. Andrew's Cross Omissions are, however, doubtless unavoidable where so much information has to be presented in so limited a compass, and in spite of all there are many years of usefulness before Pye's "Bandaging."

